# Effects of Individualized Nutrition Therapy and Continuous Glucose Monitoring on Dietary and Sleep Quality in Individuals with Prediabetes and Overweight or Obesity

**DOI:** 10.3390/nu17091507

**Published:** 2025-04-30

**Authors:** Raedeh Basiri, Yatisha Rajanala

**Affiliations:** 1 Department of Nutrition and Food Studies, George Mason University, Fairfax, VA 22030, USA; 2 Institute for Biohealth Innovation, George Mason University, Fairfax, VA 22030, USA; 3 Department of Health Administration and Policy, George Mason University, Fairfax, VA 22030, USA

**Keywords:** type 2 diabetes, prediabetes, continuous glucose monitoring, individualized nutrition therapy, dietary intake, sleep quality, alternative healthy eating index

## Abstract

**Background/Objectives:** Despite advances in public health and medical treatment, the number of patients with type 2 diabetes is increasing and it remains among the top 10 causes of death and a leading cause of disability in the United States. Early interventions with innovative approaches are essential to improving dietary intake and blood glucose control, potentially preventing or delaying type 2 diabetes and related complications. This study examined the effects of integrating real-time feedback from continuous glucose monitoring (CGM) into individualized nutrition therapy (INT) on diet and sleep quality in individuals with prediabetes and overweight or obesity. **Methods:** Thirty participants were randomized to either the treatment (*n* = 15) or the control group (*n* = 15). Both groups received individualized nutrition recommendations tailored to energy needs for weight maintenance and blood glucose control. The treatment group had real-time access to CGM data, while the control group remained blinded. Dietary intake and sleep quality were assessed using ASA24 recall and analyzed via general linear model repeated measures. **Results:** Incorporating CGM feedback into nutrition therapy significantly increased whole-grain (*p* = 0.02) and plant-based protein intake (*p* = 0.02) in the treatment group, with trends toward increased fruit intake (*p* = 0.07) and a reduced percentage of calories from carbohydrates (*p* = 0.08). Sleep efficiency also improved significantly by 5% (*p* = 0.02) following the intervention. **Conclusions:** These findings support the effectiveness of CGM-enhanced nutrition therapy in improving diet and sleep quality in individuals with prediabetes and overweight or obesity. Further research is needed to assess the sustainability and long-term impact of this approach.

## 1. Introduction

Approximately one in three American adults has prediabetes, of whom 25% will develop type 2 diabetes within three to five years [1]. Over a longer time frame, the risk of developing type 2 diabetes for individuals with prediabetes could even be as high as 70% if no interventions are made [1,2,3]. Prediabetes is a significant risk factor for developing comorbid conditions, including cardiovascular disease (CVD) [4,5,6]. Dietary interventions are known to improve insulin resistance, regulate blood glucose, and reduce the progression rate to full-blown type 2 diabetes and related complications [7,8,9,10,11,12,13,14,15,16]. The American Diabetes Association emphasizes that medical nutrition therapy (MNT) is crucial for weight loss, improved glycemic control, and reduced cardiovascular risk factors in adults with prediabetes or diabetes [17]. Current dietary recommendations for adults with prediabetes and diabetes typically prioritize low-calorie, low-carbohydrate options to support blood glucose control and weight management. However, it is essential to promote balanced eating patterns that prioritize nutrient-dense foods from all food groups in appropriate portions [18]. This approach supports long-term physical and mental health, overall well-being, and holistic disease management, offering lasting benefits beyond glucose control [19,20,21,22]. To build on the importance of balanced nutrition, it is worth noting that generalized low-carbohydrate/low-calorie diets are often not sustainable due to their restrictive nature. Studies found that only 37.5% of individuals with type 2 diabetes adhered to dietary recommendations, with factors such as family type, affordability of recommended diets, self-control regarding food, physical activity, and medication adherence significantly influencing compliance [23,24]. To achieve sustainable and effective results, dietary interventions should be personalized to align with individuals’ physiological needs and personal preferences, minimizing restrictions and maximizing adherence [25]. Individualized nutrition therapy (INT) effectively supports those with prediabetes by improving their nutritional status, which is essential for health and well-being, avoiding unnecessary dietary restrictions, and achieving or maintaining glycemic targets. It has been shown that improvements in overall diet quality decreased the risk of progression of prediabetes to type 2 diabetes [26]. Additionally, a study on the combined effect of diet quality and genetic predisposition on HbA1c levels and type 2 diabetes risk found that higher diet quality was linked to lower HbA1c levels and reduced diabetes risk, particularly in those with a higher genetic predisposition [27].

Beyond its direct impact on blood glucose management, diet quality may also enhance metabolic health by improving sleep quality. Emerging research highlights the complex relationships between prediabetes, sleep quality, and nutritional status. Short sleep duration and difficulty maintaining sleep have been linked to higher incidences of clinically identified prediabetes. Research indicates that individuals sleeping less than 5 h per day are at an increased risk of prediabetes [28,29,30]. Diet significantly influences sleep duration and quality [31,32,33]. Adherence to diets rich in fruits, vegetables, and anti-inflammatory nutrients correlates with improved sleep quality and reduced insomnia symptoms [33,34]. Additionally, diets high in simple carbohydrates and saturated fats are associated with poorer sleep quality, while diets rich in complex carbohydrates, healthy fats like unsaturated fats, and protein are linked to better sleep outcomes [35]. For those with prediabetes, poor sleep quality and suboptimal nutritional status can exacerbate insulin resistance and hinder glucose metabolism [36]; therefore, addressing both sleep and dietary habits is critical in preventing the progression of type 2 diabetes and related complications.

The early implementation of a comprehensive intervention in individuals with prediabetes is crucial to maximizing its effectiveness and increasing the likelihood of delaying the onset of type 2 diabetes. The extent to which nutritional interventions tailored to an individual’s needs and preferences can effectively improve diet and sleep quality in individuals with prediabetes remains unclear. Additionally, it is uncertain whether their effectiveness may be enhanced when combined with real-time feedback from continuous glucose monitoring (CGM). Thus, this study examined the effects of INT when combined with CGM feedback on the quality of diet and sleep in individuals with prediabetes who were overweight or obese. The primary aim of this study was to evaluate the impact of CGM-guided nutrition therapy on diet quality, as measured by Alternate Healthy Eating Index (AHEI) scores, and on the intake of individual food groups among individuals with prediabetes who were overweight or obese. The secondary aims included assessing changes in sleep duration and quality.

## 2. Materials and Methods

### 2.1. Sample Size and Power Calculations

A power analysis was conducted to determine the required sample size for detecting a statistically significant interaction between the treatment (INT vs. control) and time (across four time points). Based on an effect size of 0.25—a clinically meaningful change [37]—the sample size was calculated using G*Power Version 3.1.9.4—(Düsseldorf, Germany). The analysis indicated that at least 24 participants (12 per group) were needed to achieve 80% power at a 0.05 significance level. To account for an expected 20% dropout or missing data rate, 30 participants were ultimately enrolled.

### 2.2. Screening and Recruitment of Participants

This study was approved by the Institutional Review Board (IRB) committee at George Mason University and registered in ClinicalTrials.gov (NCT05161897). The study was advertised at George Mason University and community centers in Fairfax County. Individuals who inquired about the study were given a brief overview of the study and, if interested, pre-screened by telephone to determine eligibility before scheduling their in-person screening and visits. During the in-person screening visit, HbA1c and anthropometric measurements were performed to confirm eligibility. Eligible participants received detailed study information, and all participants signed a consent form before conducting study measurements. Participants of any race or ethnicity were eligible for the study if their ages were between 45 and 65 years, their HbA1c level was between 5.7% and 6.4%, and their BMI was between 25 and 39.9 kg/m^2^. Individuals were excluded from the study if they were pregnant or lactating women, had HbA1c and BMI outside of the inclusion ranges, or had active cancer, thyroid, kidney, liver, and/or pancreatic diseases. Those who were heavy cigarette smokers (≥25 cigarettes a day), consumed more than 12 alcoholic drinks/week on average, or had major dietary restrictions that, in the view of the investigator would limit the ability to deliver effective dietary interventions, or those who were participating in any weight loss or dietary program, taking prescribed appetite suppressants or weight loss medications, or participating in another investigational study concurrently were also excluded from the study.

### 2.3. Study Design

A total of 30 eligible individuals who consented to this study were randomly assigned to either the treatment (*n* = 15) or control (*n* = 15) group, using an online random number generator. At baseline, anthropometric measurements were conducted, and both groups were asked about their medical and medication history. Participants were asked to complete a 24-h dietary recall using the Automated Self-Administered 24-Hour (ASA24^®^) Dietary Assessment Tool [38] at baseline, and subsequently on the day of each visit. Participants were also asked to record all food and beverage consumption throughout the study using a food diary. This information was used for educational purposes during the study for the treatment group and after the study for the control group. Participants wore CGM devices throughout the study (30 days). The treatment group had access to their real-time blood glucose data on their cell phones, while the control group remained blind to these data. During the initial 10-day period, participants were instructed to maintain their usual dietary habits. This allowed us to establish consistent baseline measurements of dietary intake and behavior across all participants before beginning the intervention. Participants visited the lab every 10 days over the 30-day study period to replace their CGMs, receive dietary guidance, and consult with a dietitian. Both groups received nutrition education and had the opportunity to ask questions directly to a dietitian during the second and third visits. The 20-day intervention period was designed to provide participants with sufficient time to implement dietary changes and observe their effects on blood glucose levels using CGM data [39]. 

### 2.4. Study Interventions

During the second visit, all participants met with a registered dietitian to receive dietary recommendations tailored to their energy needs for weight maintenance, calculated using the Mifflin–St. Jeor equation [40]. A moderate-carbohydrate diet (50% carbohydrate, 20% protein, 30% fat), as recommended by the American Diabetes Association [41], was prescribed for all participants. The rationale for selecting this diet was that the specified macronutrient distribution has been shown to effectively improve blood glucose levels and reduce cardiovascular disease risk factors. The diabetic exchange list [42] was utilized to educate participants about food groups and offer flexibility in selecting food items aligned with their personal preferences. Both groups received guidance on recommended serving sizes for each food group, carb choices, and the appropriate amount of carbohydrates per meal [43]. However, only the treatment group received additional personalized feedback based on their CGM data during the second and third visits. The dietitian reviewed food diaries with each participant in the treatment group and, using CGM data, highlighted foods that caused blood glucose spikes. This information was then used to set individualized goals aimed at reducing the frequency of high blood glucose events. The control group did not receive CGM-based feedback or personalized goal setting based on glucose patterns.

### 2.5. Anthropometric Measurements

Height was measured without shoes using a wall-mounted stadiometer, weight was assessed with a digital scale (Health o meter^®^ Professional Scales, McCook, IL, USA), and body mass index (BMI) was calculated using the following formula: BMI = weight (kg)/[height (m)^2^].

### 2.6. Calculating the Alternative Healthy Eating Index (AHEI) Score

The AHEI score was calculated based on dietary intake variables extracted from the ASA24 [38]. The scores were computed by evaluating 10 dietary components [44]; specifically, vegetables, fruits, whole grains, sugar-sweetened beverages and fruit juices, nuts and legumes, red and processed meats, Long-chain n–3 fatty acids, polyunsaturated fats, sodium, and alcohol, with each component assigned a score ranging from 0 to 10. Higher scores indicate better adherence to healthy dietary patterns and a lower risk of chronic diseases such as cardiovascular disease, type 2 diabetes, and all-cause mortality [44,45].

### 2.7. Assessing Sleep Efficiency

Participants used ASA24 to record key sleep parameters such as time of sleeping, how long it took to fall asleep, final wake-up time, and total awake time [46]. Participants recorded their sleep times in a 24-h format, which was later converted into total minutes for analysis. The duration of a sleep episode (DSE) was calculated using the following formula [47]: Duration of sleep episode (DSE) = Sleep onset latency (SOL) + Total sleep time (TST) + Wake after sleep onset (WASO) + Time attempting to sleep after final awakening (TASAFA); where SOL refers to the time taken to fall asleep after turning the lights off, TST represents the total duration of sleep scored, WASO is the total time awake after initial sleep onset but before the final awakening, and TASAFA accounts for time spent trying to return to sleep after multiple wake-ups.

Furthermore, sleep efficiency (SE) was calculated as the proportion of time spent asleep relative to the total duration of the sleep episode using the following formula: SE = (TST/DSE) × 100 [47].

### 2.8. Statistical Analysis

Data analysis was performed using the Statistical Package for the Social Sciences (SPSS) version 29.0 (SPSS, Inc., Chicago, IL, USA), with statistical significance set at *p* < 0.05. Descriptive statistics were used to evaluate population characteristics, and an analysis of variance (ANOVA) table assessed covariate distribution across groups. Significant differences in potential confounding variables between groups were controlled by including those variables as covariates in the model. Changes in AHEI scores, the dietary intake of each food group, and sleep duration and efficiency throughout the study were evaluated using a general linear model with repeated measures.

## 3. Results

A total of 30 participants were recruited from Fairfax County and the surrounding area. The mean age ± SD for participants in the treatment and control groups was 57.3 ± 5.2 y and 52.7 ± 6.4 y, respectively. The distribution of potential covariates, including sex, race, age, and baseline BMI, was assessed between the groups. Only age showed a significant difference (*p* = 0.04) and was therefore included as a covariate in the analysis models. The detailed demographic characteristics of the participants are presented in Table 1.

### 3.1. Dietary Modifications and AHEI Score Changes

At baseline, the mean AHEI scores for both groups were notably low, with no significant differences observed between the groups. We noticed a significant interaction between baseline BMI and AHEI; therefore, we included it in the model as a covariate. The AHEI score was improved for the treatment group from 49.9 to 52.8, while it was slightly increased from 49.9 to 50.5 in the control group. None of the changes in the groups were statistically significant.

### 3.2. Changes in Total Calorie Intake (Kcal)

The control group showed slightly higher calorie intake compared to the treatment group at baseline; however, the difference between the groups was not statistically significant. During the study, the treatment group showed a numerical reduction in calorie intake from 1774 to 1688 kcal, while the control group decreased from 2148 to 2092 kcal.

### 3.3. Changes in Macronutrient Distribution

The treatment group decreased their percentage of calorie intake of carbohydrates from 38.6% to 34.0%, while the control group showed a smaller reduction from 37.1% to 36.0%. Although both groups showed a decrease, the reduction in the treatment group was more consistent and trended toward statistical significance (4.6%, *p* = 0.08), whereas the change in the control group (1.1%) was not significant. Additionally, the treatment group increased their percentage of calorie intake of protein by 1.9% (20.1 to 22.0%), compared to only 0.1% in the control group (20.7 to 20.8%). Similarly, the treatment group increased their calorie intake from fats by 2.7% (38.2% to 40.9%), while the control group experienced a smaller increase of 1.1% (37.8% to 38.9%).

### 3.4. Changes in the Intake of Fiber, Sugar, Cholesterol, and Fat Types

**Figure 1:**
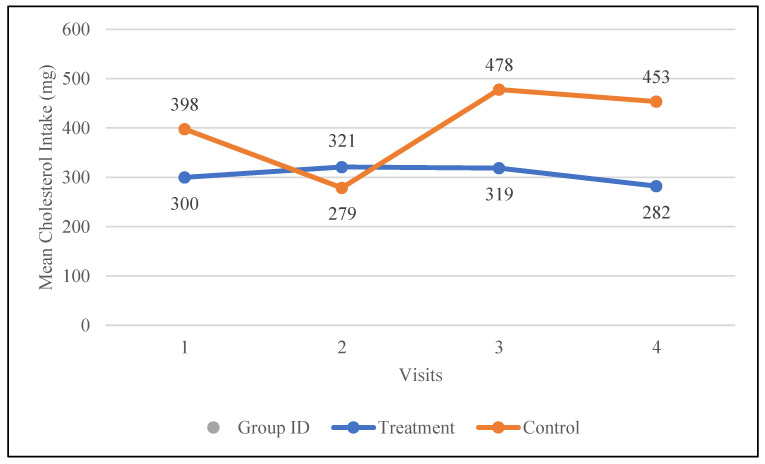
Changes in the mean total cholesterol intake (mg) in the control and treatment groups over the 30-day study period. Participants were visited at baseline and every 10 days thereafter, for a total of four visits.

Fiber intake increased by 1.9 g in the treatment group, rising from 17.9 g to 19.8 g, whereas it decreased by 4.7 g in the control group, dropping from 22.5 g to 17.8 g. Sugar intake was reduced by 12.9 g in the treatment group, whereas it increased by 30 g in the control group. Although not statistically significant, changes in the treatment group support better health, while those in the control group have a negative impact. The mean total saturated fat intake increased in both the treatment group (21.4 to 24.5 g) and the control group (28.0 g to 30.0 g); however, these changes were not statistically significant. The intake of polyunsaturated fats increased following the nutrition intervention in both groups but declined again by visit four. These changes were not statistically significant. Cholesterol intake was decreased in the treatment group from 300 to 282 mg and increased from 398 to 453 mg in the control group. Figure 1 shows changes in the dietary intake of cholesterol for both groups.

### 3.5. Assessing Dietary Intake from Different Food Groups

#### 3.5.1. Fruits

**Figure 2:**
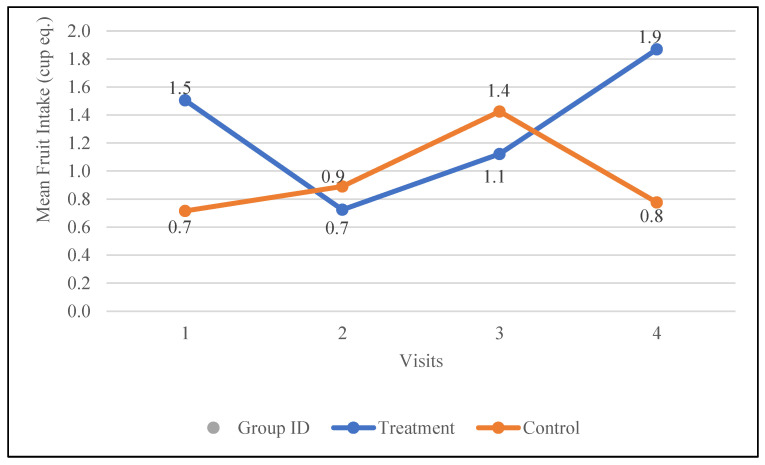
Changes in the mean fruit intake (cup equivalents) in the control and treatment groups over the 30-day study period. Participants were visited at baseline and every 10 days thereafter, for a total of four visits.

Fruit intake was notably low at baseline for both the treatment group (1.5 ± 0.3 cup eq.) and the control group (0.7 ± 0.3 cup eq.). Following the intervention, fruit intake increased by 0.4 cup eq. in the treatment group, compared to a smaller increase of 0.1 cup eq. in the control group. Notably, fruit intake in the treatment group consistently increased after receiving nutrition education, rising from 0.7 cup eq. at visit two to 1.9 cup eq. at visit four. In contrast, the control group showed an initial increase from visit one to visit three (0.7 to 1.4 cup eq.), followed by a decline to 0.8 cup eq. at visit four. The changes in fruit intake in the treatment group following the intervention showed a trend toward statistical significance (*p* = 0.07). The changes in the fruit intake for both groups are depicted in Figure 2.

#### 3.5.2. Vegetables

At baseline, the mean total vegetable intake was 2.2 cup eq. in the treatment group and 1.7 cup eq. in the control group. During the study, vegetable intake increased to 2.7 cup eq. in the control group but decreased to 2.0 cup eq. in the treatment group. However, these changes were inconsistent in both groups and did not reach statistical significance.

#### 3.5.3. Whole Grains

**Figure 3:**
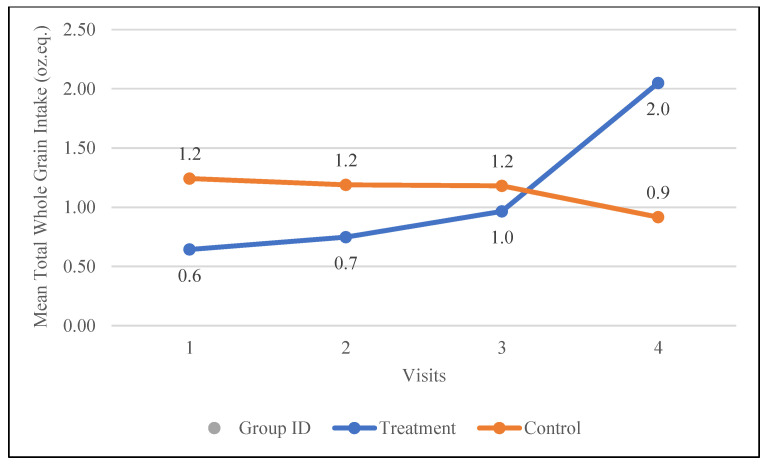
Changes in the mean whole-grain intake (oz. equivalents) in the control and treatment groups over the 30-day study period. Participants were visited at baseline and every 10 days thereafter, for a total of four visits.

The treatment group showed a significant increase in whole-grain intake, rising by 1.4 oz. eq. (from 0.6 to 2.0 oz. eq., *p* = 0.02), while the control group experienced a slight decrease of 0.3 oz. eq. (from 1.2 to 0.9 oz. eq.). As illustrated in Figure 3, the treatment group demonstrated a consistent upward trend in whole-grain intake throughout the study, whereas the control group displayed a steady, non-statistically significant decline.

### 3.6. Total Protein Food Intake

**Figure 4:**
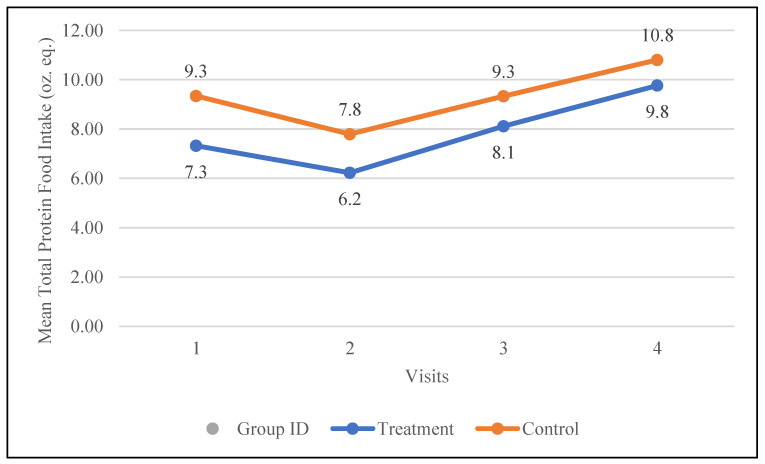
Changes in the mean total protein intake (oz. equivalents) in the control and treatment groups over the 30-day study period. Participants were visited at baseline and every 10 days thereafter, for a total of 4 visits.

The control group had a higher baseline intake of total protein foods (9.3 oz. eq.) compared to the treatment group (7.3 oz. eq.). Following the intervention, both groups increased their total protein food intake; however, the treatment group demonstrated a larger increase of 2.5 oz. eq. compared to 1.5 oz. eq. in the control group. The changes in the mean total protein food intake for both groups are demonstrated in Figure 4.

#### 3.6.1. Total Protein Intake from Animal Sources

**Figure 5:**
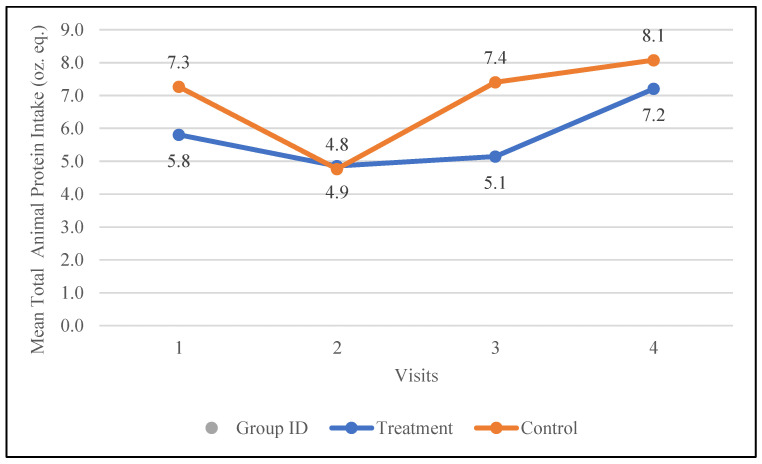
Changes in the mean total animal protein intake (oz. equivalents) in the control and treatment groups over the 30-day study period. Participants were visited at baseline and every 10 days thereafter, for a total of four visits.

Changes in the total protein intake from animal sources, i.e., meat, poultry, seafood, organ meat, and cured meat (oz. eq.), were examined during the study. The treatment group increased their intake of animal-based protein from 5.8 oz. eq. to 7.2 oz. eq., while the control group increased their intake from 7.3 oz. eq. to 8.1 oz. eq. The changes in the participants’ protein intake from animal-based protein are depicted in Figure 5.

#### 3.6.2. Total Protein Intake from Plant Sources

The changes in total protein intake from soy, nuts, seeds, and legumes were calculated as protein intake from plant sources. During the study, the treatment group increased their protein intake from plant sources by 0.6 oz. eq., while the control group decreased their intake by 1.0 oz. These changes were statistically significant within the treatment group (*p* = 0.02) following the intervention, but did not reach significance when compared between groups.

### 3.7. Dairy Products

**Figure 6:**
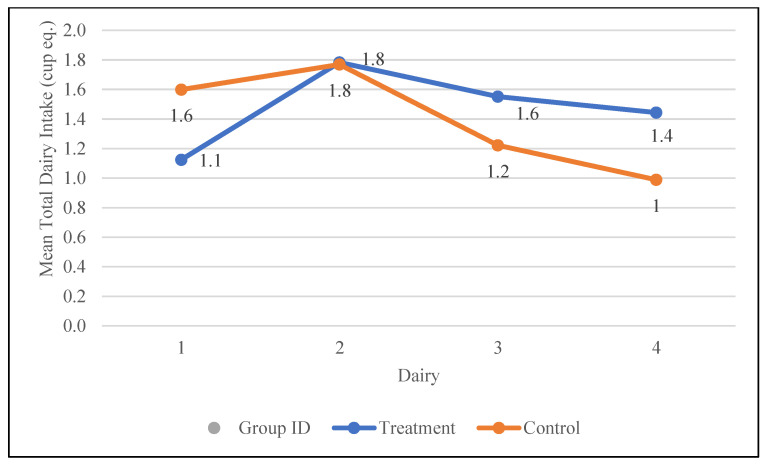
Changes in the mean total dairy intake (cup equivalents) in the control and treatment groups over the 30-day study period. Participants were visited at baseline and every 10 days thereafter, for a total of four visits.

Total milk, yogurt, cheese, and whey intake were also assessed during the study. The differences between the groups were not statistically significant over the duration of the study. However, the total dairy intake increased from 1.1 to 1.4 cup eq. in the treatment group and decreased from 1.6 to 1 cup eq. in the control group. The changes in the dairy intake of participants during the study are depicted in Figure 6.

### 3.8. Sleep Duration and Quality

At baseline, there were no significant differences in sleep duration (409 vs. 394 min) or sleep efficiency (83% vs. 85%) between the intervention and control groups, respectively. Over the course of the study, sleep duration remained relatively stable in the treatment group (409 to 408 min) but decreased notably in the control group (394 to 358 min). Sleep efficiency improved by 5% in the treatment group (83% to 88%, *p* = 0.02) but numerically declined by 2% in the control group (85% to 83%).

## 4. Discussion

Overall, our results showed that adding CGM feedback to INT could better enhance the effects of nutrition therapy in improving the quality of diet and sleep in individuals with prediabetes and overweight or obesity. This study aimed to improve the quality of diet and did not focus on restricting calorie intake. The findings of this study confirmed good compliance with participants, as the average calorie intake of the groups remained within the recommended range during the study. The mean AHEI score was improved by 3 points in the treatment group but only by 0.6 points in the control group. A systematic review and meta-analysis found that higher AHEI scores were associated with a 22% lower risk of type 2 diabetes and cardiovascular disease [48]. Moreover, a large prospective study examining the interaction between genetic risk and diet quality on type 2 diabetes risk found that a 10-unit decrease in the AHEI score was associated with a 13% higher risk of developing type 2 diabetes (HR: 1.13, 95% CI: 1.09–1.17) [49]. Therefore, the improved AHEI score in the treatment group, in addition to the notable decrease in percent calorie intake from carbohydrates, a decrease in sugar intake, an increase in protein intake particularly from plant sources, and a decrease in cholesterol in the treatment group suggests reduced risk in progression to type 2 diabetes and CVD [50].

In both groups, the mean fiber intake was significantly lower than the recommendations by the Academy of Nutrition and Dietetics (AND) at baseline. The intervention improved the fiber intake in the treatment group while it decreased in the control group. Although the mean fiber intake still did not reach the recommended amount by AND at the end of the study, the observed changes suggest additional health benefits in the treatment group. The treatment group consistently decreased their sugar intake during the study, while the control group notably increased theirs, albeit not statistically significantly. Changes in fruit intake tended to be significant in the treatment group after receiving nutrition intervention; however, this was not observed in the control group, resulting in a significant difference in fruit intake between the groups at the end of the study (*p* = 0.04). Both groups consistently increased their intake of protein foods after receiving nutrition therapy. When we separated protein food intake based on the source (animal vs. plant), the treatment group showed an increase in their intake from both animal and plant sources by 1.4 and 0.8 oz. eq., respectively, while the control group increased their intake only from animal sources by 0.8 oz. eq. but decreased their intake of plant protein foods by 1.1 oz. eq. Several studies suggest that increasing plant protein intake may offer protective benefits against the progression of prediabetes to type 2 diabetes [51,52,53]. Regarding dairy products, both groups decreased their intake after intervention; however, the decrease was more notable in the control group (0.8 cup eq.) compared to the treatment group (0.4 cup eq.). Evidence from cohort and meta-analytic studies suggests that, in adults with pre-diabetes, higher intakes of yogurt and other dairy—particularly low-fat milk/yogurt and even moderate whole-fat dairy—are consistently linked to a lower risk of progressing to type 2 diabetes or a greater likelihood of reverting to normoglycemia [54,55,56,57].

The more notable improvement in diet quality, observed in the treatment group, underscores the potential benefits of integrating CGM into nutrition therapy for individuals with prediabetes. This approach may be particularly beneficial for those with prediabetes who have overweight or obesity and struggle with achieving or maintaining weight loss with the current weight loss diets [58]. The treatment group showed more favorable changes in AHEI scores, macronutrient distribution, and the dietary intake of food groups compared to the control group. However, statistical significance was not reached for some outcome variables, likely due to the short 20-day intervention. It has been shown that even with intensive and structured lifestyle interventions, significant dietary changes could only be reached after several months to one year [59]. The observed improvements in diet quality among participants in the treatment group were accompanied by significantly better blood glucose management compared to the control group. Our previous analysis showed a significant increase in the percentage of time spent within the target blood glucose range (*p* = 0.02), as well as significant reductions in mean blood glucose concentration (*p* < 0.05), the glucose management indicator (*p* = 0.02), percent coefficient of variation for blood glucose (*p* = 0.01), and percent of time spent in high or very high blood glucose ranges (*p* = 0.04) in the treatment group [60]. These changes were not statistically significant in the control group, suggesting that the improvements in diet quality observed in this study may have contributed to better glycemic control in the treatment group. Therefore, the observed improvements in diet quality, even if not statistically significant, suggest a promising approach for enhancing blood glucose management in individuals with prediabetes. Future clinical studies with larger sample sizes and longer intervention durations are needed to better evaluate the long-term impact of this approach.

The findings also showed that sleep efficiency was significantly improved in the treatment group by 5%; however, it declined in the control group by 2%. Research indicates that poor sleep quality is associated with impaired glucose metabolism and increased insulin resistance. A narrative review found that sleep education programs improved sleep quality and reduced HbA1c levels in type 2 diabetes patients [61]. Additionally, a meta-analysis found that both short and long sleep durations, as well as poor sleep quality, are linked to higher hemoglobin A1c levels [62], a marker of long-term blood glucose control, in patients with type 2 diabetes. Therefore, this approach may contribute to improved blood glucose management by enhancing sleep quality as well.

The strength of this study is that, to our knowledge, this is the first study to evaluate the effects of adding CGM feedback to INT in order to examine their potential effects on diet and sleep quality in individuals with prediabetes and overweight or obesity. Although 24-h dietary recalls can introduce reporting bias, we sought to reduce this by encouraging participants to immediately log their daily food and beverage intake in a food diary. The treatment group was informed that their detailed dietary records would be reviewed at each visit alongside CGM data to tailor personalized dietary recommendations. Meanwhile, the control group was told that their dietary intake would be assessed at the study’s conclusion to provide feedback on foods impacting their blood glucose levels. This approach ensured a consistent level of effort across groups. We believe these strategies equipped participants with both a practical tool and motivation to report their intake as accurately as possible. We also had a diverse population, which is important for the generalizability of the data. However, this study also had some limitations that should be considered, including the small sample size and short duration of intervention. Future clinical studies with larger sample sizes and longer intervention durations are thus needed to better evaluate the long-term impact of this approach.

## 5. Conclusions

This study demonstrated that incorporating CGM feedback into individualized nutrition therapy could enhance improvements in both diet and sleep quality in individuals with prediabetes and overweight or obesity. Therefore, this approach may serve as an effective strategy to support better blood glucose management and sleep quality in this population.

## Figures and Tables

**Table 1:** Demographic characteristics of participants and distribution of potential covariates at baseline.

Group	Treatment	Control	*p*-Value
Sex (male/female)	11/4	11/4	1.00
Race (White/Black/Asian)	9/1/5	10/2/3	
Live alone (yes/no)	1/14	2/13	0.55
Employed (yes/no)	14/1	12/3	0.07
Financial concerns (yes/no)	11/4	12/3	0.7
HbA1c * (%)	5.7 ± 0.78	6.0 ± 0.2	0.26
BMI ** (kg/m^2^)	31.8 ± 4.3	31.4 ± 4.5	0.84

* hemoglobin A1c. ** Body Mass Index.

## Data Availability

The datasets used and/or analyzed during the current study are available from the corresponding author upon reasonable request.
